# Prevalence and patterns of multimorbidity among mining workers of Odisha, India: a cross-sectional study

**DOI:** 10.3389/fpubh.2025.1613856

**Published:** 2025-08-19

**Authors:** Abhinav Sinha, Md Shaney Ali, Mark P. Funnell, Arohi Chauhan, Patrick J. Highton, Srikanta Kanungo, Sanghamitra Pati

**Affiliations:** ^1^ICMR-Regional Medical Research Centre, Bhubaneswar, India; ^2^Diabetes Research Centre, Leicester General Hospital, University of Leicester, Leicester, United Kingdom; ^3^National Institute for Health and Care Research Applied Research Collaboration East Midlands, Leicester, United Kingdom; ^4^South Asian Institute of Health Promotion, Bhubaneswar, India

**Keywords:** multimorbidity, mining workers, India, multiple long-term conditions, occupational health, health promotion

## Abstract

**Introduction:**

Multimorbidity is an emerging public health challenge in India due to rapid industrialization, urbanization and an aging population. Multimorbidity often impacts occupational outcomes, including work-related stress, job loss, absenteeism, and reduced years of service. Odisha, an eastern state of India, is a major mining state with a large workforce exposed to occupational physical and mental stress, and toxic waste. We determined prevalence of multimorbidity and assessed its correlates among mining workers in the Odisha, India. Additionally, we assessed the most common patterns of chronic conditions in this group.

**Methods:**

A cross-sectional study was conducted among 425 mining workers aged ≥18 years in Sukinda, Odisha, from January to July 2022. Participants were recruited using systematic random sampling. A pre-validated multimorbidity Assessment Questionnaire for Primary Care was used for face to face interviews following standardized protocols. Multivariable logistic regression models assessed associations between multimorbidity and socio-demographic characteristics. A matrix analysis identified common dyads and triads of chronic conditions.

**Results:**

The overall prevalence of multimorbidity was 37.41%. The most common dyad was acid peptic disease + chronic backache (10.06%), while the most frequent triad was acid peptic disease + chronic backache + chronic chest pain (1.89%). Irregular exercise [AOR: 4.66 (95% CI: 1.74–12.49)], and longer service in the mining industry (31–40 years) [AOR: 8.05 (95% CI: 1.91–33.86)] were significantly associated with multimorbidity.

**Conclusion:**

The high prevalence of multimorbidity among mining workers highlights the urgent need for workplace health policies and/or interventions prioritizing ergonomic improvements, chronic disease management, and routine health screenings.

## Introduction

While improvements in living conditions and healthcare have extended life expectancy, they have also led to a rise in non-communicable diseases (NCDs) globally (1). Consequently, the coexistence of chronic infectious diseases and NCDs in India, similar to trends in other low-and middle-income countries (LMICs), creates a complex healthcare landscape, where individuals must increasingly manage multiple long-term conditions, a phenomenon known as multimorbidity (2). Multimorbidity defined as the concurrent presence of two or more chronic conditions regardless of the primary underlying disease (2) is associated with increased healthcare utilization, expenditure and premature mortality. Furthermore, individuals with multimorbidity often report poorer perception of their physical and mental health; decreased quality of life; and reduced functional capacity (3).

Multimorbidity is emerging as an important public health challenge in India. A study conducted in Odisha, India, found the prevalence of multimorbidity to be around 28.3% among primary care patients, ranging from 5.8% in patients aged 18 to 29 years to 45% in those aged ≥70 years and above, a prevalence comparable to that observed in high-income countries (4). A recent systematic review estimated the prevalence of multimorbidity in India to be ~20% (5). Much of this burden can be attributed to rapid industrialization, urbanization, physical inactivity and an ageing population (6). Multimorbidity also has a significant impact on occupational outcomes, including increased work-related stress, job loss, absenteeism, presenteeism and fewer years of service (7–9). Evidence suggests a strong link between multimorbidity and work incapacity due to multiple illnesses (10).

In India, the mining industry is a prominent sector, employing approximately 0.9 million workers as of 2021 (11). The sector involves both metallic and non-metallic resource extraction and has long been regarded as one of the world’s most hazardous workplaces due to the high incidence of occupational diseases, injuries, and fatalities (12, 13). Odisha is among the richest mining states in India, boasting the largest natural resources, including 32.9% of the nation’s iron ore reserves, as well as vast amounts of bauxite (60.0%), chromite (98.4%), coal (24.8%), and manganese (67.6%) (14). The Sukinda region of Odisha, which contains approximately 98% of India’s chromite ores, accounts for 99% of the total chromite production (14). Despite this economic significance, Odisha’s mineral-rich regions are classified as aspirational districts (i.e., part of the Aspirational Districts Programme (ADP) which aims to improve the socio-economic status of certain districts in India) (11, 12). Workers in these areas face significant health risks due to prolonged exposure to toxic waste, combined with physical and mental stress (15).

Given these health risks, we conducted a study in the Sukinda Valley region of Odisha, focusing on workers in chromite mining sites. This study aimed to determine the prevalence of multimorbidity, and assess its key correlates, among mining workers in the Sukinda Valley region of Odisha. Additionally, we assessed common occurring patterns of chronic conditions among these individuals.

## Methods

### Study design and setting

A cross-sectional study was conducted among the mining workers in the Sukinda region of Odisha, India, from January 2022 to July 2022. Sukinda is located in northern Odisha, a region in Eastern India. Ten operational chromite mining sites were purposively selected. Permissions were obtained from local authorities to interview the mining workers in the valley region for this study.

### Study population

The participants were selected from ten different mining locations who were actively involved in mining activities for any length of time. Participants aged ≥18 years, who provided written informed consent were included in the study. Pregnant women and individuals unwilling to participate were excluded.

### Sample size and sampling technique

The prevalence of multimorbidity was estimated to be 28% based on previous literature (4). With a 95% confidence interval and 5% significance level, a minimum sample size of 310 miners was required. Accounting for a 10% non-response rate, the minimum required sample was 340. We recruited 425 participants, ensuring sufficient statistical power to estimate multimorbidity prevalence with desired precision. This oversampling was both feasible and intentional, as many mining workers expressed strong interest in participating, viewing the study as an opportunity to better understand their health status. Additionally, oversampling helped reduce sampling variability and facilitated a more comprehensive understanding of patterns of co-occurring conditions.

Participants were recruited using systematic random sampling. At the end of each work shift, every third worker present on-site was approached with a participant information sheet. After explaining the purpose and requirements of the study, workers who provided written informed consent were interviewed. If a selected worker (i.e., every third individual) declined to participate, the next eligible worker was approached to maintain the sampling interval. This approach helped maintain the integrity of the systematic random sampling method and ensured the representativeness of the study sample. We achieved a high response rate of 93.8%, with 425 out of 453 eligible workers participating in the study.

### Data collection and management

The Multimorbidity Assessment Questionnaire for Primary Care (MAQ-PC) was used for the survey (16). The MAQ-PC is a validated tool developed through an iterative process and has been widely used in primary care and community-based surveys across multiple LMICs. The questionnaire included sections on socio-demographic profiles and the presence of self-reported chronic conditions from a list of 26 diseases. Face to face interviews were conducted using a mobile-based application called Open Data Kit Collect (ODK) on Android phones. To ensure uniformity, data collectors were trained following a standardized protocol. Additionally, a quality control protocol was implemented, including random checks of 10% of the data by investigators to ensure accuracy.

### Data analysis

Data was downloaded in Microsoft Excel format from the server and checked for discrepancies. Analysis was performed using Stata v. 17.0 (StataCorp, Texas). Multimorbidity was assessed by summing the number of chronic conditions, with individuals reporting two or more chronic conditions classified as having multimorbidity. Descriptive statistics were presented as frequencies and proportions. A 95% confidence interval (CI) was calculated for all proportions as a measure of uncertainty. Chi-square test was conducted to assess bivariate associations prior to logistic regression. A bivariate logistic regression model was used to assess associations between multimorbidity and socio-demographic characteristics, with the strength of association expressed as odds ratio (OR) and 95% CI. A multivariable logistic regression model was also conducted reported as adjusted odds ratio (AOR) and 95% CI. A simplistic matrix technique was used to analyze all feasible and exhaustive combinations of chronic conditions with a frequency of more than 1% to identify patterns of co-occurring conditions (dyads and triads) (18).

### Ethical considerations

Ethical approval was obtained from the Institutional Human Ethics Committee of ICMR-Regional Medical Research Centre, Bhubaneswar. Additionally, necessary permissions were secured from the Municipal Corporation and management of the mining sites. All participants provided informed written consent before participation.

## Results

The mean age of the participants was 38.7 ± 10 years ranging from 18 to 59 years. Most of the participants were males (98%) and were from deprived (36%) or middle-class (37%) backgrounds. Almost one-third of the workers did not participate in any physical activity. Most participants were native to the region and lived in rural communities (Table 1).

**Table 1:** Socio-demographic profile and prevalence of multimorbidity across various socio-demographic characteristics among study participants.

Attributes	Category	Total (*N* = 425) *n* (%)	Multimorbidity *n*, % (95% CI)
Age (years)	18–30	105 (24.7)	18, 17.1 (10.4–25.7)
	31–40	150 (35.3)	57, 38 (30.2–46.2)
	41–50	99 (23.2)	43, 43.3 (3.4–53.7)
	51–60	71 (16.7)	41, 57.7 (45.4–69.3)
Gender	Male	419 (98.5)	158, 37.7 (33–42.5)
	Female	6 (1.4)	1, 16.6 (0.4–64.1)
Ethnicity*	Scheduled caste (SC)	29 (6.8)	12, 41.3 (23.5–61)
	Scheduled tribe (ST)	46 (10.8)	22, 47.8 (32.8–63)
	Other backward class (OBC)	131 (30.8)	49, 37.4 (29.1–46.2)
	General	219 (51.5)	76, 34.7 (28.4–41.4)
Marital status	With partner	377 (88.7)	141, 37.4 (32.4–42.5)
	Without partner	48 (11.2)	18, 37.5 (23.9–52.6)
Migrant status	Migrant	16 (3.7)	8, 50 (24.6–75.3)
	Native	409 (96.2)	151, 36.9 (32.2–41.7)
Residence	Rural	390 (91.7)	145, 37.1(32.3–42.1)
	Suburban/Peri-urban	11 (2.5)	4, 36.3 (10.9–69.2)
	Urban	24 (5.6)	10, 41.6 (22.1–63.3)
Physical activity	Sometimes	238 (56)	79, 33.1 (27.2–39.5)
	Regularly	36 (8.4)	10, 27.7 (14.2–45.1)
	Never	151 (35.5)	70, 46.3 (38.2–54.6)
Wealth quintile	Poor	153 (36)	56, 36.6 (28.9–44.7)
	Middle	159 (37)	45, 28.3 (21.4–35.9)
	Rich	113 (26)	58, 51.3 (41.7–60.8)
Health insurance	Public scheme	234 (55)	82, 35 (28.9–41.5)
	Private scheme	15 (3.5)	9, 60 (32.2–83.6)
	No health insurance	176 (41.4)	68, 38.6 (31.4–46.2)
Years in service	<5	72 (16.9)	14, 19.4 (11.0–30.4)
	6–10	75 (17.6)	23, 30.6 (20.5–42.3)
	11–20	159 (37.4)	65, 40.8 (33.1–48.9)
	21–30	90 (21.1)	37, 41.1 (30.8–51.9)
	31–40	29 (6.8)	20, 68.9 (49.1–84.7)

^*^SC, socially disadvantaged and discriminated groups recognized for affirmative action and welfare; ST: indigenous communities based on specific criteria such as geographic isolation, distinctive culture, and socio-economic disadvantage, eligible for affirmative action; OBC, socially and educationally disadvantaged communities; General, individuals who do not belong to other categories.

The overall prevalence of multimorbidity was 37.41% (95% CI: 32.91–42.13). The prevalence of multimorbidity increased with age. Individuals from urban areas had a higher prevalence of multimorbidity. Additionally, multimorbidity was more common among those who did not engage in physical activity.

The commonly occurring combination of two chronic conditions (dyad) was acid peptic disease + chronic backache (10.06%), hypertension + chronic backache (3.77%), and chronic backache + obesity (3.14%). The most frequent triad was acid peptic disease + chronic backache + chronic chest pain (1.89%). A detailed description of these patterns is presented in Table 2.

**Table 2:** Patterns of commonly occurring chronic conditions.

Patterns	*n*	%
Dyads		
Acid peptic disease + Chronic back ache	16	10.06
Hypertension + Chronic back ache	6	3.77
Chronic back ache + Obesity	5	3.14
Hypertension + Acid peptic disease	5	3.14
Acid peptic disease + Obesity	4	2.52
Diabetes + Hypercholesterolemia	4	2.52
Diabetes + Acid peptic disease	4	2.52
Hypertension + Diabetes	4	2.52
Chronic back ache + Chronic chest pain	3	1.89
Chronic back ache + Eczema	3	1.89
Chronic back ache + Migraine	3	1.89
Acid peptic disease + Migraine	3	1.89
Acid peptic disease + Piles	3	1.89
Diabetes + Obesity	3	1.89
Acid peptic disease + Eczema	2	1.26
Acid peptic disease + Psoriasis	2	1.26
Acid peptic disease + Arthritis/Rheumatism/Osteoporosis/Other joint disease	2	1.26
Chronic respiratory disease (Asthma/emphysema/COPD*/Bronchitis) + Acid peptic disease	2	1.26
Hypertension + Chronic constipation	2	1.26
Triads		
Acid peptic disease + Chronic back ache + Chronic chest pain	3	1.89
Chronic back ache + Hypercholesterolemia + Obesity	2	1.26
Acid peptic disease + Chronic back ache + Obesity	2	1.26
Acid peptic disease + Chronic back ache + Dry cough	2	1.26
Acid peptic disease + Arthritis/Rheumatism/Osteoporosis/Other joint disease + Obesity	2	1.26
Acid peptic disease + Arthritis/Rheumatism/Osteoporosis/Other joint disease + Chronic Back ache	2	1.26
Diabetes + Hypercholesterolemia + Obesity	2	1.26
Diabetes + Acid Peptic Disease + Obesity	2	1.26
Diabetes + Acid Peptic Disease + Sleep Disorder	2	1.26
Diabetes + Acid peptic disease + Oral conditions (Dental caries/bleeding or swelling gums/loose teeth)	2	1.26

^*^COPD: Chronic Obstructive Pulmonary Disease.

The likelihood of multimorbidity increased with age, with the highest odds observed among individuals aged 51–60 years [AOR: 7.11 (95% CI: 2.38–21.17)], followed by those in the 40–50 years age group [AOR: 4.90 (95% CI: 1.90–12.65)]. Participants belonging to Scheduled Tribe category had higher odds [AOR: 3.38 (95% CI: 1.50–7.63)] of having multimorbidity (Table 3). Individuals without a partner were at higher risk [AOR: 3.24 (95% CI: 1.33–7.92)] of multimorbidity than those with a partner. Irregular physical exercise [AOR: 4.66 (95% CI: 1.74–12.49)] was also associated with a higher likelihood of developing multimorbidity. Additionally, workers with longer service in the mining industry (31–40 years) had significantly higher odds of multimorbidity [AOR: 8.05 (95% CI: 1.91–33.86)].

**Table 3:** Logistic regression analysis depicting association of multimorbidity with various socio-demographic characteristics.

Variables	Category	Odds ratio [95% CI]	Adjusted odds ratio [95% CI]
Age (years)	18–30	Reference	Reference
	31–40	2.96 [1.61–5.42]	3.45 [1.53–7.79]
	41–50	3.71 [1.94–7.07]	4.90 [1.90–12.65]
	51–60	6.60 [3.30–13.20]	7.11 [2.38–21.17]
Ethnicity	Scheduled caste	1.32 [0.60–2.92]	1.80 [0.73–4.47]
	Scheduled tribe	1.72 [0.90–3.27]	3.38 [1.50–7.63]
	Oher backward classes	1.12 [0.71–1.76]	1.19 [0.69–2.03]
	General	Reference	Reference
Marital status	With partner	Reference	Reference
	Without partner	1.00 [0.53–1.86]	3.24 [1.33–7.92]
Physical activity	Sometimes	1.29 [0.59–2.81]	4.66 [1.74–12.49]
	Regularly	Reference	Reference
	Never	2.24 [1.01–4.98]	2.54 [0.98–6.55]
Wealth quintile	Poor	Reference	Reference
	Middle	0.68 [0.42–1.10]	0.47 [0.25–0.87]
	Rich	1.82 [1.11–2.99]	1.08 [0.55–2.12]
Years in service	<5	Reference	Reference
	6–10	1.83 [0.85–3.92]	2.88 [1.10–7.50]
	11–20	2.86 [1.47–5.56]	3.67 [1.40–9.58]
	21–30	2.89 [1.40–5.93]	1.95 [0.63–6.02]
	31–40	9.20 [3.45–24.51]	8.05 [1.91–33.86]

## Discussion

The prevalence of multimorbidity was high among mining workers, with chronic backache emerging as one of the most common chronic conditions across dyads and triads. Multimorbidity was significantly associated with age, belonging to Scheduled Tribe caste, having a partner, irregular physical activity, and longer service in the mining industry.

The prevalence of multimorbidity among mining workers was approximately 37%, closely aligning with findings from two systematic reviews reporting rates of multimorbidity to be 36.4% in LMICs and 37.2% globally (17, 18). However, the emergent pattern of chronic conditions among mining workers differs markedly from those in the general population likely due to prolonged exposure to dust and hazardous fumes, physically demanding and repetitive tasks, machinery vibrations, and factors such as inadequate nutrition and irregular meal patterns, as noted by Alagarajan et al. (5). Notably, the 37% prevalence among miners is substantially higher than the 20% pooled prevalence reported in a meta-analysis of Indian studies, and far exceeds the 2.4% prevalence found among working-age individuals in Odisha (19).

This disparity underscores the need for a systematic assessment of chronic conditions among mining workers to ensure continuity of care. Notably, the Mines Act of 1952 includes provisions for periodic health evaluations of mining workers, a six-day work schedule, and free treatment for medically unfit individuals, making the healthcare more accessible and affordable for this population (20).

Acid peptic disease + chronic backache was the most common dyad, while acid peptic disease + chronic backache + chronic chest pain was the most frequently observed triad in this study. In contrast to the multimorbidity clusters identified in prior systematic reviews, which highlight three main groups—cardiovascular and metabolic diseases, mental health disorders, and musculoskeletal conditions (21)—our examination of mine workers indicated a notably high prevalence of musculoskeletal disorders. This predominance may be attributed to the demanding, physically intensive nature of mining tasks, which involve heavy lifting, repetitive actions, and long periods of working in ergonomically challenging settings. These job-related factors can lead to the emergence of musculoskeletal issues, which may be less evident in the broader population. While existing literature from India frequently indicates that cardio-metabolic and gastrointestinal disorders are prevalent among adults in general community settings (22–24), the distinctive working conditions in mining may suggest a shift in this trend toward musculoskeletal disorders. This variation may highlight the importance of customizing occupational health policies to reflect the specific exposure risks associated with mining environments. Multimorbidity was significantly associated with age, which is consistent with the findings of a systematic review of longitudinal studies, where age was identified as a major risk factor for multimorbidity (25). Additionally, individuals belonging to Scheduled Tribe groups were found to be at higher risk, aligning with a systematic review that reported indigenous populations were twice likely to experience multimorbidity compared to their non-indigenous counterparts (26). The increased risk may be attributed, in part, to factors like reduced use of prescribed medications and limited access to quality healthcare services within Scheduled Tribe groups, leading to delays in diagnosis and treatment. Also, cultural traditions and geographic isolation worsen these inequalities, amplifying the overall burden of multimorbidity in this demographic. Furthermore, an analysis of the Indian SAGE dataset also observed a higher prevalence of multimorbidity among tribal older adults (27).

Irregular physical activity was identified a risk factor for multimorbidity, this is unsurprising given that low physical activity levels are a known contributor to multimorbidity risk (28). A study from India similarly found that lack of physical activity was associated with higher multimorbidity prevalence among older adults (29). This highlights the importance of behavioral change communication, and potentially workplace health promotion, to adopt healthy lifestyle practices in this population.

Moreover, this study observed that longer service in the mining industry was associated with higher multimorbidity risk beyond the effect of aging, which aligns with the findings of a systematic review that reported multimorbidity as a significant factor negatively impacting work (30). This association may be due to cumulative effects of aging, prolonged exposure to physically and mentally demanding mining work, and extended contact with occupational hazards like dust and toxic fumes. These factors can accelerate the development of chronic conditions, even after accounting for age (31). Multimorbidity reduces quality of life and productivity, increases absenteeism and presenteeism rates, raises the likelihood of temporary or permanent work leave, and lowers employability and workforce participation.

### Implications for policy and practice

We observed a high prevalence of multimorbidity (~37%) among mining workers, with distinct patterns of chronic conditions primarily musculoskeletal and gastrointestinal disorders. These findings highlight the need for focused workplace health strateges, including ergonomic interventions, chronic illness management initiatives, and routine health screenings. Preventive measures such as promoting physical activity and structured health education could be beneficial, given the observed association between multimorbidity and longer service duration in the mining industry.

Given the increased risk among Scheduled Tribe communities, healthcare policies should aim to improve equitable access to medical care and social support for vulnerable populations. While our study did not assess compliance with the Mines Act, 1952, strengthening its particularly provisions related to regular health assessments may enhance early detection and management of chronic conditions. Finally, integrating multimorbidity care into existing occupational health initiatives could support employee well-being, reduce absenteeism, and improve productivity. These recommendations are based on observed health needs and warrant further exploration in future research and policy evaluations.

### Strengths and limitations

Key strength of this study includes the utilization of the Multimorbidity Assessment Questionnaire for Primary Care, a pre-validated instrument specifically created to evaluate multimorbidity. Additionally, the study’s large sample size and inclusion of diverse age groups enhance the applicability of the results. Furthermore, gathering data from an at-risk demographic like mine workers is a notable strength, offering novel perspectives on multimorbidity within this susceptible population.

On the other hand, dependence on self-reported chronic illnesses may result in an underrepresentation of the actual prevalence of multimorbidity. Further, the study did not assess the severity or functional impact of individual conditions or disease combinations, limiting insights into their clinical burden. Given that the study adopts a cross-sectional approach, it is not capable of determining causal links. Subsequent studies should aim to corroborate self-reports with clinical evaluations and utilize longitudinal frameworks to gain deeper insights into causal pathways within this group.

## Conclusion

The prevalence of multimorbidity was high (~37%) among mining workers from the Sukinda region of Odisha, India, with chronic backache emerging as one of the most common chronic conditions. The high prevalence of multimorbidity among mining workers underscores the urgent need for targeted workplace health policies that prioritize ergonomic improvements, chronic disease management programs, and routine health screenings.

## Data Availability

The raw data supporting the conclusions of this article will be made available by the authors, without undue reservation.
